# Observations and conversations: how communities learn about infection risk can impact the success of non-pharmaceutical interventions against epidemics

**DOI:** 10.1186/s12889-021-12353-9

**Published:** 2022-01-05

**Authors:** Matthew J. Silk, Simon Carrignon, R. Alexander Bentley, Nina H. Fefferman

**Affiliations:** 1 Centre for Ecology and Conservation, University of Exeter Penryn Campus, Penryn, UK; 2 Environment and Sustainability Institute, University of Exeter, Exeter, UK; 3 National Institute for Mathematical and Biological Synthesis (NIMBioS), University of Tennessee, Knoxville, TN USA; 4 Center for the Dynamics of Social Complexity (DySoC), University of Tennessee, Knoxville, TN USA; 5 Department of Anthropology, University of Tennessee, Knoxville, TN USA; 6 School of Information Science, University of Tennessee, Knoxville, TN USA; 7 Department of Ecology and Evolutionary Biology & Mathematics, University of Tennessee, 447 Hesler Biology Building, Knoxville, TN 37996 USA

**Keywords:** Multilayer networks, Behavioral epidemiology, Coupled human-natural systems

## Abstract

**Background:**

Individual behavioural decisions are responses to a person’s perceived social norms that could be shaped by both their physical and social environment. In the context of the COVID-19 pandemic, these environments correspond to epidemiological risk from contacts and the social construction of risk by communication within networks of friends. Understanding the circumstances under which the influence of these different social networks can promote the acceptance of non-pharmaceutical interventions and consequently the adoption of protective behaviours is critical for guiding useful, practical public health messaging.

**Methods:**

We explore how information from both physical contact and social communication layers of a multiplex network can contribute to flattening the epidemic curve in a community. Connections in the physical contact layer represent opportunities for transmission, while connections in the communication layer represent social interactions through which individuals may gain information, e.g. messaging friends.

**Results:**

We show that maintaining focus on awareness of risk among each individual’s physical contacts promotes the greatest reduction in disease spread, but only when an individual is aware of the symptoms of a non-trivial proportion of their physical contacts (~ ≥ 20%). Information from the social communication layer without was less useful when these connections matched less well with physical contacts and contributed little in combination with accurate information from physical contacts.

**Conclusions:**

We conclude that maintaining social focus on local outbreak status will allow individuals to structure their perceived social norms appropriately and respond more rapidly when risk increases. Finding ways to relay accurate local information from trusted community leaders could improve mitigation even where more intrusive/costly strategies, such as contact-tracing, are not possible.

## Background

Our current best public health recommendations for mitigation of the COVID-19 pandemic rely on using behavioural interventions such as social distancing and mask wearing, and behaviourally driven acceptance of vaccines (where available) to curtail transmission of infection. The success of these policies requires widespread adherence to achieve epidemic control; as with herd immunity, threshold effects in efficacy mean that gaps in adoption can quickly compromise any benefits [1, 2]. Therefore, identifying how the adoption of these behaviours is shaped over the course of an epidemic is a key challenge in designing effective mitigation strategies [3–6].

Adherence, however, relies on individual behavioural choices and so can be complicated to understand and predict [3, 7]. Well-established theory from psychology acknowledges that the factors influencing whether or not people take action are complicated [8, 9]. Many theories of behaviour and behaviour change have been applied to understanding health behaviour [10]. One of the dominant theories (the theory of planned behaviour [11]), posits that action is an outcome of interaction between an individual’s attitudes and beliefs, their perception of social norms regarding that behaviour, and their perception of their own behavioural control over their actions (alternative theories of behaviour, such as Value-Belief-Norm theory [12] also posit similar influences, though in different relation to each other). In the case of COVID-19, adoption of and adherence to behavioural interventions are therefore likely to be predicated on perception of two main features: a) individual attitudes and beliefs about personal risk of infection and its consequences [13], and b) the social norms around adherence in the individual’s community [14]. Over time the changing attitudes and beliefs within each person’s network will drive complex, non-linear dynamics in population-level behaviours [15–17].

An individual’s perception of these features is shaped by communication within their network of friends, neighbours, and community leaders [18, 19]. Most likely, the network of a person’s close physical contacts, through which they risk infection, differs from their regular communication network (in person and online) of people who contribute to their attitudes and beliefs surrounding preventative behaviours, and from whom they are likely to estimate the social norms of their community. These distinct networks underlie a disconnect between someone’s perception of their risk versus their actual risk. On one hand, an individual’s communication network could provide early warning of encroaching exposure risks derived from the spread of awareness ahead of the infection itself [20, 21]. On the other hand, the mismatch between the communication and infection networks may mean that an individual could underestimate their risk (e.g., communication networks are likely to be more sparsely connected than networks of infection-relevant contacts in populations that are not socially distancing). Despite this, we still understand relatively little about the potential implications of acquiring information from these two different sets of contacts.

The dynamics triggered by the spread of awareness through the population are further complicated by the timescales of observable risk due to the etiology of COVID-19. The latency in the development of symptoms and the capacity for presymptomatic, or even asymptomatic, transmission make estimation of real-time risk by surveillance complicated, even without considering different sources of information [22]. In terms of understanding disease prevalence, the relative reliance of individuals in shaping their beliefs, and thus their actions, on their own direct observation of health among their daily physical contact network may have an effect that is distinct from that of their (potentially more geographically distant) communication network. The balance of these distinct network effects may therefore be the critical feature in determining the success of behavioural public health measures to combat COVID-19.

**Fig. 1:**
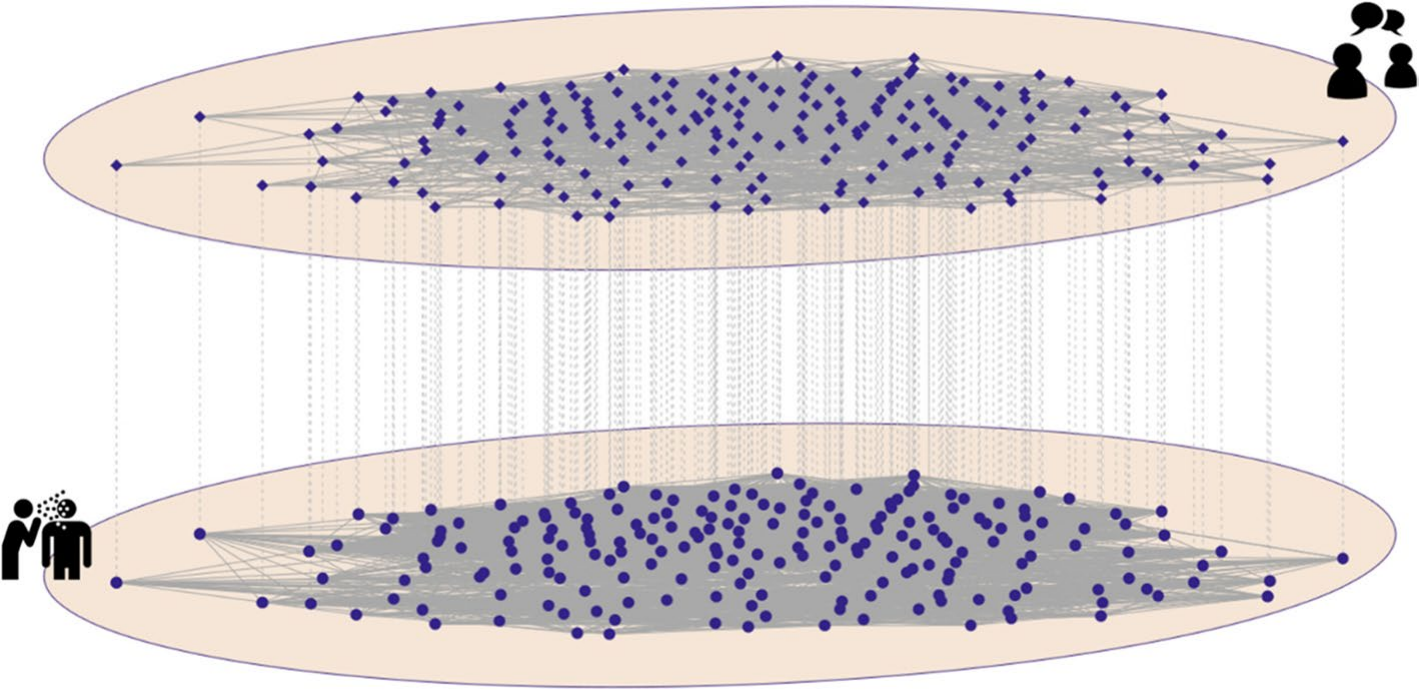
Illustration of part of a multiplex network from our coupled behavioural-epidemiological models. Multiplex networks enable the representation of distinct, but potentially overlapping, types of social interactions between the same set of individuals. Here we show the communication layer (top) and infection layer (bottom) from one community (200 of 2000 nodes) within one of our nine multiplex networks. We illustrate the first community from the multiplex network with a relative modularity of 0.6 in both layers and no homophily in either layer

Multiplex networks have commonly been used to quantify complex patterns of social relationships in human societies, including the incorporation of off- and online social ties [23–26]. Multiplex networks treat different sets of interactions as separate layers within a multilayer network object (Fig. 1). Intra-layer edges reflect different types of interaction (e.g. close contact, online communication etc.) and inter-layer edges can only connect the same individual in different layers [27]. Individuals can be connected in any number of layers, allowing sets of connections to overlap. By considering multiple, dependent sets of social connections multiplex networks have proved a valuable tool in epidemiological modelling [28–30]. We employ a theoretical multiplex network model, implemented stochastically, to test the relative adoption of behavioural interventions in populations of individuals who rely on a) their communication network layer only (henceforth referred to as simply the “communication layer”), b) their physical contact network layer only (henceforth referred to as the “infection layer”), and c) both layers simultaneously to inform their understanding of COVID-19, and therefore their individual adherence to protective behaviours such as mask wearing or social distancing. We further consider the influence of structure in both layers of the network and how that structure might impact the behaviour of populations as they rely on perceptions constructed from contacts in those layers. Geographic and social heterogeneity in contact structure are modelled using different levels of modularity (i.e. differences in local versus global density of contacts; see Methods). We also consider the potential impact homophily based upon predisposition (defined here to be a characteristic or belief that may influence the tendency of individuals to be socially connected) in either the communication or both layers of the network. While not exhaustive, these studies offer insight into how communities can reinforce the types of informational access that foster protective behavioural decision making among their members.

## Methods

### Overview

We used stochastic, theoretical models to test how the awareness of symptomatic neighbours in either a) the set of people that a person who communicates with on a regular basis (their communication layer), b) the set of people that a person is in close proximity to (their infection layer), or c) both of these sets of contacts can impact epidemic spread of an infection with COVID-19 like dynamics. We simulated realistic (but not data-driven), multiplex social networks for our populations that coupled a layer of infection-relevant contacts through which the epidemic was simulated and a communication layer through which concern about the disease could simultaneously spread (see Fig. 1). All modelling was conducted in R3.6.1 [31] and the code used is provided on GitHub (https://github.com/matthewsilk/CoupledDynamics2_layeruse). The general modelling framework was the same as that used by Silk et al. [15] and is additionally described in that paper and in the Supplementary Material.

### Population generation

We generated populations of 2000 individuals (a balance between minimising stochasticity in early epidemic outcomes and computational efficiency), which consisted of children (24%), young adults (63%) and older adults (13%). Age classes could differ in the social connections, epidemiological outcomes and concern about the disease (as detailed below). Individuals also had one of two baseline predispositions and homophily by predisposition impacted patterns of social connections (in either or both layers of the multiplex network).

### Social network generation

We used the same 9 multiplex social networks as detailed in Silk et al. [15]. These were coupled, multiplex networks that connected all individuals within a communication layer that influenced the spread of concern about the disease and an infection layer that influenced the transmission of the pathogen itself (Fig. 1). A full description of the algorithm used to generate these networks is provided in the Supplementary Material. For this study, global edge densities were always higher in the infection layer than in the communication layer. The network contained either a) no homophily in either layer, b) homophily in the communication layer, or c) homophily in both layers. Community structure was introduced using a re-wiring algorithm (as detailed in the Supplementary Material): either the relative modularity of both layers was set to 0.4, both to 0.6, or the infection layer was set to 0.6 and the communication layer to 0.4. Each child was assigned two parents from the same predisposition and community. If children shared one parent they also shared the other but parents could be connected or unconnected. Each young adult formed connections with a number of older adults of the same predisposition (representing older relatives, friends or community members) as detailed in the Supplementary Material. Children shared the same connections to older adults as their parents. When the multiplex network was constructed we re-assigned parents from the infection layer to match those in the communication layer. Child-older adult connections were re-assigned accordingly.

### Concern model

We used the same concern model as Silk et al. [15]. Concern about the disease was modelled as a complex contagion [32] through the communication layer. Whether an individual was adherent to mitigation measures or not (a binary trait) was based on a Bernoulli draw in which the probability of adherence depended on an underlying trait continuous we term concern. While this simplifying assumption is reasonable for a suite of overlapping protective behaviours, individuals can vary in their adherence to different protective behaviours in reality [33–35]. As a result, individuals could fluctuate between adherent or non-adherent states and this was more likely if they had intermediate values of concern. Concern could be influenced by a) Social Construction (becoming more concerned if neighbours in the communication layer were adherent), b) Reassurance (becoming less concerned if all neighbours in the communication layer were perceived to be healthy (i.e. symptom free) and c) Awareness (becoming more concerned if network neighbours were symptomatic). For this study the information gained for Awareness could be gained from either the communication layer, the infection layer or both layers. Because an individual is unlikely to find out about the status of every individual in their infection layer, we conducted additional simulations in which there was imperfect detection of symptomatic contacts in the infection layer (probability of detection: 0.5, 0.2 and 0.05). We tested 10 values for the strength of the Awareness Effect per day per symptomatic network connection (0, 0.1, 0.2, 0.4, 0.6, 0.8, 1, 1.2, 1.4 and 1.6). Values of the Social Construction Effect and Reassurance Effect were drawn from uniform distributions (between 0 and 0.5 and between − 0.1 and 0 respectively). We selected these parameter values based on our previous model [15]. The concern of children was not modelled. They were assigned as adherent if either or both of their parents were concerned.

Each time an individual became adherent they cut connections to a negligible edge weight with a 50% probability ( [36]; see Supplementary Material) within the infection layer while maintaining their connectivity in the communication layer. If an individual became non-adherent then these edge weights returned to their full weight.

### Infectious disease model

Our infectious disease model is a very similar stochastic network model to that described in Silk et al. [15] with etiological parameter values adapted from [37, 38] and adjusted to match empirical data from the COVID-19 pandemic. Briefly, we adjusted the transmission probability so that, in the absence of behavioural change, approximately 80% of our population would be infected by the epidemic, and set daily probabilities of hospitalisation and death so that outcomes in our model closely approximated those seen during the pandemic (see 15 for further details). The model contains susceptible (S), exposed (E), pre- or mildly symptomatic (I1), symptomatic (I2), hospitalised (I3), recovered (R) and dead (D) compartments. Parameter values are provided in Table S1 and details of the algorithm used are provided in the Supplementary Material. The transition from S to E depended on the number of contacts a susceptible individual had with infected individuals (I1, I2, I3) with a pre-defined probability of transmission per contact (see Table S1). Ill (I2) and hospitalised (I3) individuals cut all their connections in the infection layer to 0.001, meaning that individuals are only likely to spread infection when in compartment I1. The length of time individuals spent in the compartments E, I1, I2 and I3 was drawn from a Poisson distribution with means provided in Table S1. Individuals in the I2 compartment has a daily probability of transitioning to I3 which was dependent on their age (see Supplementary Material). Individuals in I3 had an age-dependent daily probability of dying (see Supplementary Material).

### Simulations

For this paper we conducted simulations for the nine multiplex networks described (with different combinations of homophily by predisposition and modularity), for 50 values of the Social Construction and Reassurance Effects (paired draws from independent uniform distributions) and 10 values of the Awareness Effect. We then conducted simulations in which the Awareness Effect (observational learning of symptomatic infection) applied to a) contacts in the communication layers, b) contacts in the infection layer and c) contacts from both layers combined. For scenario b) we repeated the full set of simulations with 0.5, 0.2 and 0.05 probability of symptomatic contacts being detected at each day. This resulted in a total of 27,000 independent simulation runs. In each simulation, individuals were allocated initial values of concern whereby 20% of the adult population would be expected to be adherent at the start of the simulation. For each simulation run we simulated a maximum time period of 300 days or stopped simulations when no individuals were in the E, I1, I2 or I3 compartments. The simulation algorithm was similar to that used in Silk et al. [15] and is detailed in the Supplementary Material.

### Analysis

To compare between different runs of the simulations we quantified the height of the epidemic peak at a population level by aggregating the daily counts of symptomatic infections in all 10 communities. This measure of the height of the epidemic peak indicated how successfully each simulated population managed to “flatten the curve” with their adherence to mitigating behaviours [4]. We compare epidemic peaks from when individuals learned about symptomatic network neighbours from different types of social contact while considering values of the Social Construction and Reassurance Effects. To help explain some of the differences between the infection and communication layers in their ability to “flatten the curve” we also examined the similarity of connections in these layers by quantifying the proportion of contacts in each layer that were also present in the other for each multiplex network.

## Results

**Fig. 2:**
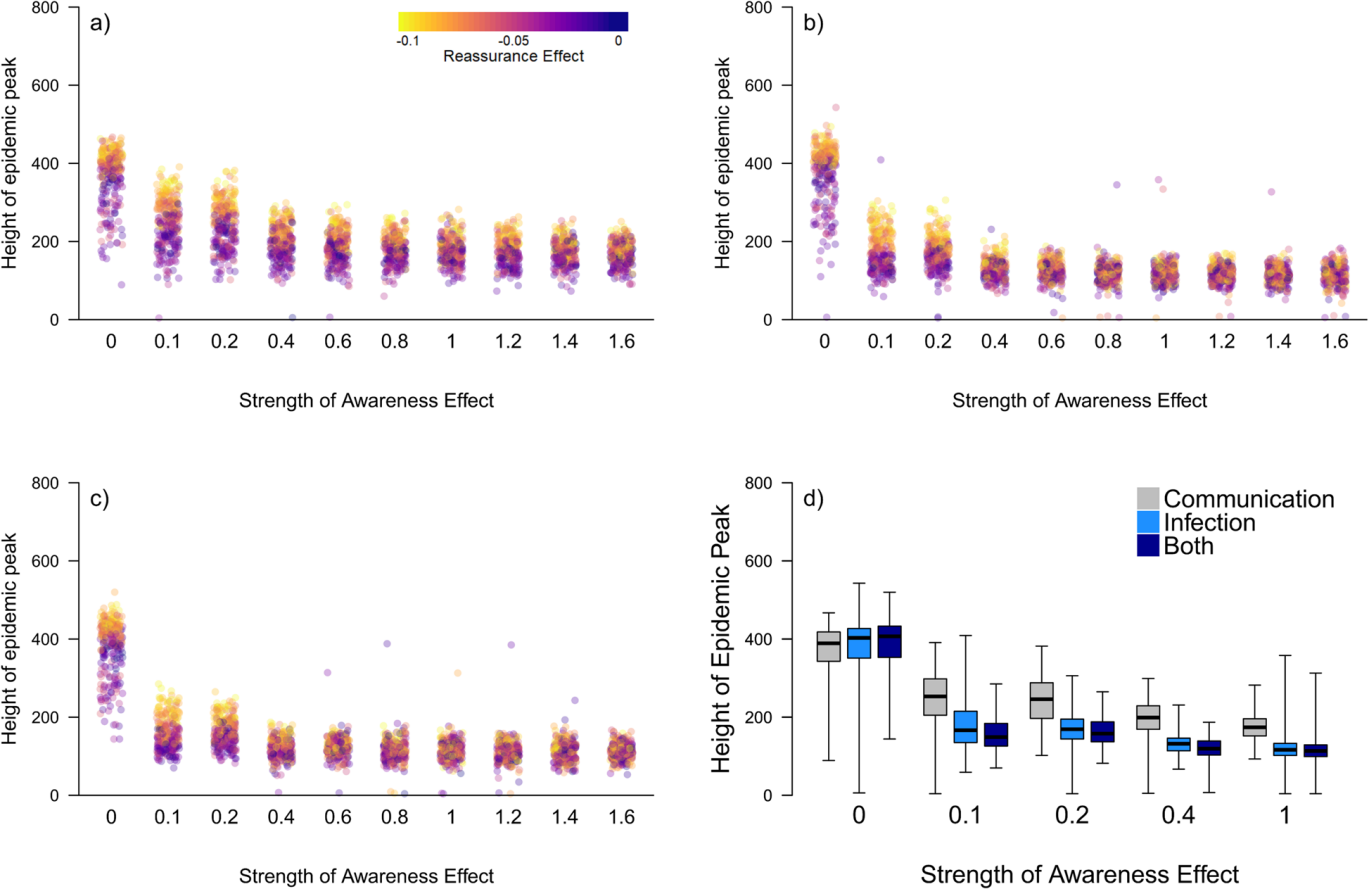
The relationship between the height of the epidemic peak (i.e. maximum simultaneous number of symptomatic [I2] individuals) and strength of the Awareness Effect (unitless; the extent to which individuals become more concerned for each symptomatic network neighbour they have; see Supplementary Material) when Social Construction is weak (< 0.3). An individual learns of symptomatic contacts from **a**) their communication layer, **b**) their infection layer (learning from 100% of contacts) and **c**) both layers together. The colour of points in panels (**a**-**c**) indicates the strength of the Reassurance Effect: yellow indicating a strong Reassurance Effect through to purple indicating a weak Reassurance Effect. In panel **d**) we contrast the height of the epidemic peak directly for selected values of the Awareness Effect. Boxes indicate the interquartile range, the bold horizontal line the median and the whiskers extend to the full range of the data

**Fig. 3:**
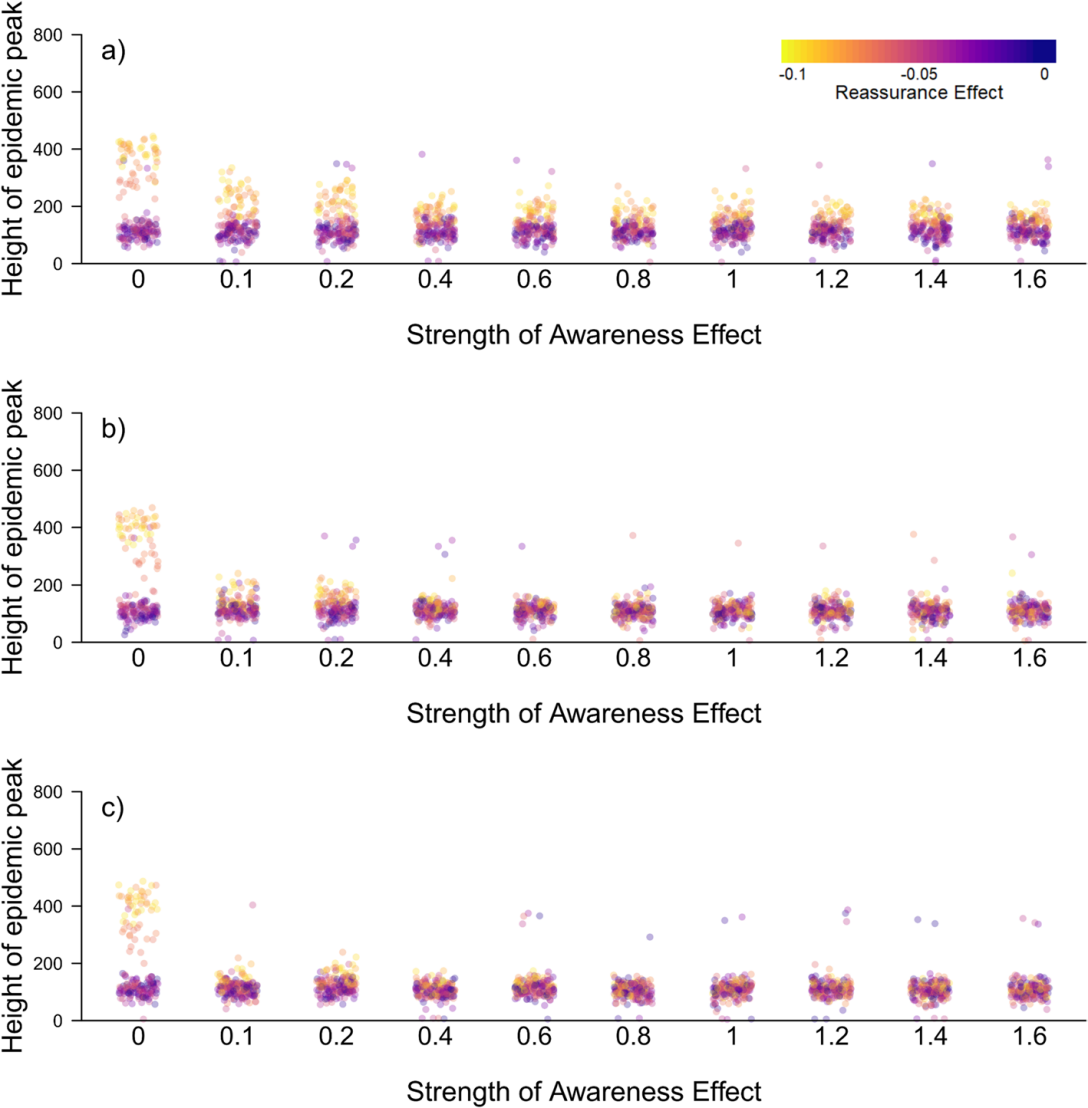
The relationship between the height of the epidemic peak (i.e. maximum simultaneous number of symptomatic [I2] individuals) and strength of the Awareness Effect (unitless; the extent to which individuals become more concerned for each symptomatic network neighbour they have; see Supplementary Material) when Social Construction is strong (> 0.3). An individual learns of symptomatic contacts from **a**) their communication layer, **b**) their infection layer (learning from 100% of contacts) and **c**) both layers together. The colour of points indicates the strength of the Reassurance Effect: yellow indicating a strong Reassurance Effect through to purple indicating a weak Reassurance Effect

When we assume an individual can identify 100% of symptomatic contacts, the Awareness Effect is more effective in flattening the curve when people respond to illness in their infection layer rather than in their communication layer (panels a) versus b) in Figs. 2 and 3). When this is the case, even relatively weak Awareness Effects can contribute to flattening the curve. Using information from the infection layer alone is nearly as effective as using information from both the infection and communication layers except when the Awareness Effect is very weak (compare panels b) and c) in Figs. 2 and 3). When social construction is weak there is an important difference, regardless of the strength of the Reassurance Effect (Fig. 2). When social construction is instead strong, it plays an important role in flattening the curve except in the case when the Reassurance Effect is also strong, meaning that differences caused by the source of information for the Awareness Effect are only noticeable when this is the case (Fig. 3). Consequently, we focus on the case when Social Construction is weak for subsequent results.

A second notable difference that arises when people respond to prevalence in their infection layer rather than the communication layer is that the strength of the Reassurance Effect becomes less important. When individuals respond to illness in their communication layer, the epidemics always have higher peaks with a strong Reassurance Effect (also reliant on the communication layer) even when the Awareness Effect is strong and the curve has been flattened (Fig. 2a). However, when the Awareness Effect is stronger (> 0.6), learning about illness from the infection layer or both layers results in similar epidemic peaks regardless of the strength of the Reassurance Effect (Fig. 2b and c).

**Fig. 4:**
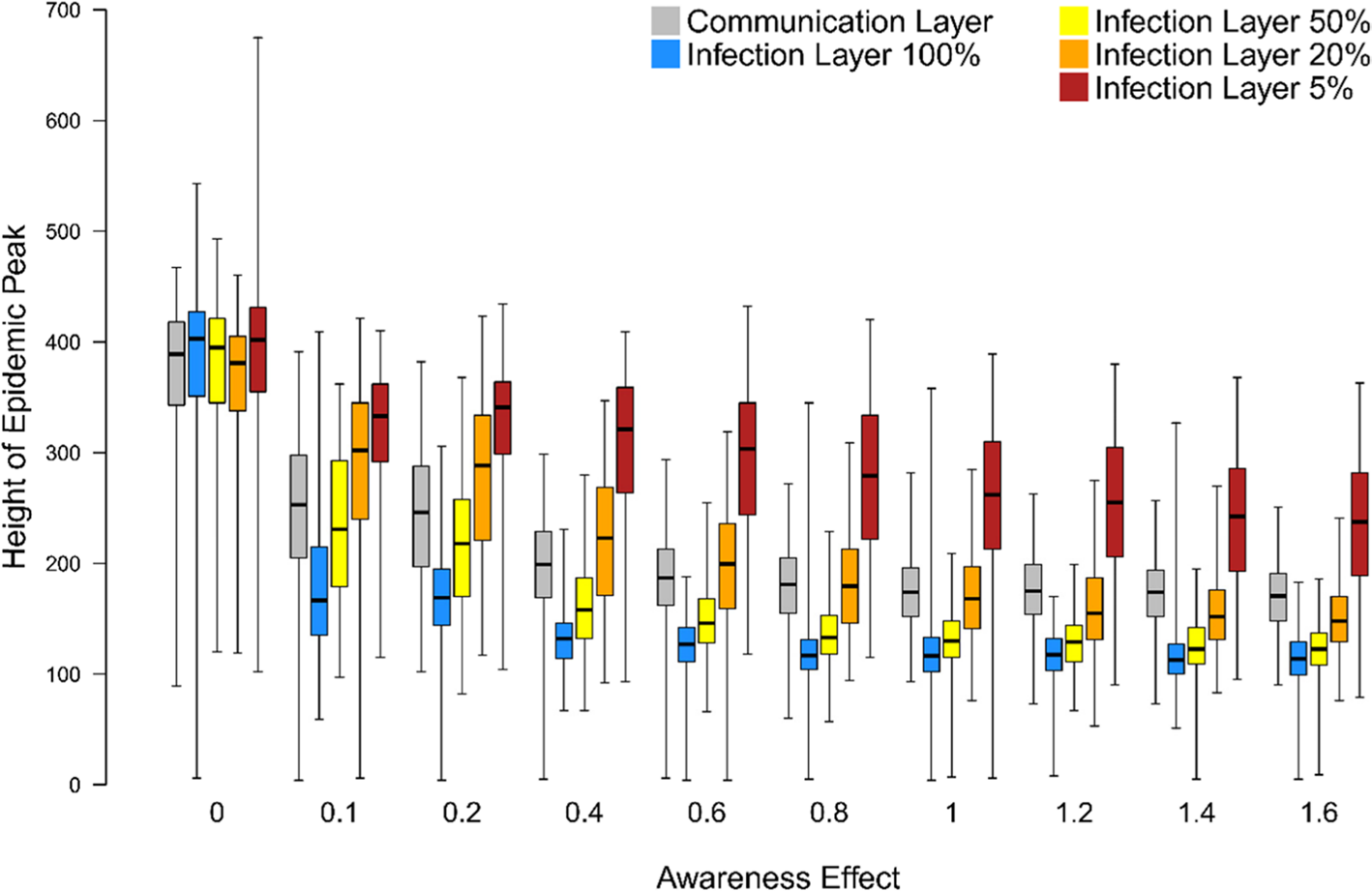
The relationship between the height of the epidemic peak (i.e. maximum simultaneous number of symptomatic [I2] individuals) and strength of the Awareness Effect (unitless; the extent to which individuals become more concerned for each symptomatic network neighbour they have; see Supplementary Material) when Social Construction is weak (< 0.3). We show the relationship when Awareness is acquired through the communication layer (grey) and the infection layer when 100% (blue), 50% (yellow), 20% (orange) and 5% (red) of symptomatic contacts are detected each day. Boxes indicate the interquartile range, the bold horizontal line the median and the whiskers extend to the full range of the data

When we assume that individuals can partially identify the symptomatic contacts in their infection layer, the mitigating effect is reduced considerably in our networks (Fig. 4). When there is a 50% chance of an individual detecting an ill neighbour in their infection layer, the height of the epidemic peak remains lower than when an individual gains information on the prevalence of infection from their communication layer, with the difference increasing as the Awareness Effect gets stronger. When there is a 20% chance of detection in the infection layer, the epidemic peak is marginally higher than when (accurate) information is used from the communication layer with a weak Awareness Effect and slightly lower with a strong Awareness Effect. When there is a 5% chance of detection the mitigating influence of the Awareness Effect is very limited indeed and restricted to strong Awareness Effects.

**Fig. 5:**
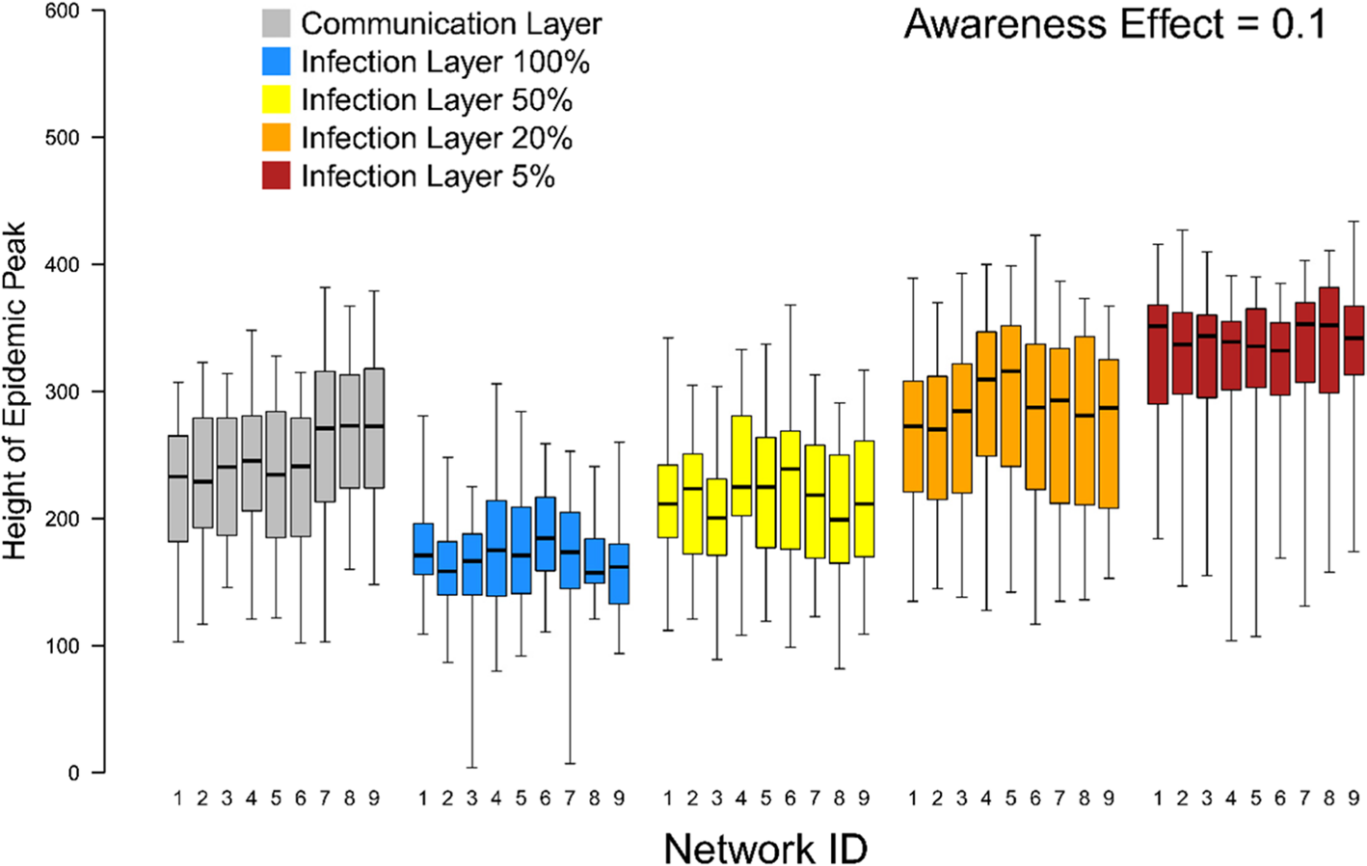
The relationship between the height of the epidemic peak and how an individual finds out about symptomatic contacts when the Social Construction Effect is weak (< 0.3) for an Awareness Effect of 0.1 plotted separately for each of the nine multiplex networks used in the study. Networks 1-3 have no homophily in either layer, networks 4-6 have homophily in both layers and networks 7-9 have homophily in the communication layer only. In networks 1, 4 and 7 both layers have a relative modularity of 0.4, in networks 2, 5 and 8 both layers have a relative modularity of 0.6, and in networks 3, 6 and 9 the relative modularity of the infection layer is 0.6 and the relative modularity of the communication layer is 0.4. Plots for other Awareness Effects our qualitatively similar (see Fig. S1)

**Fig. 6:**
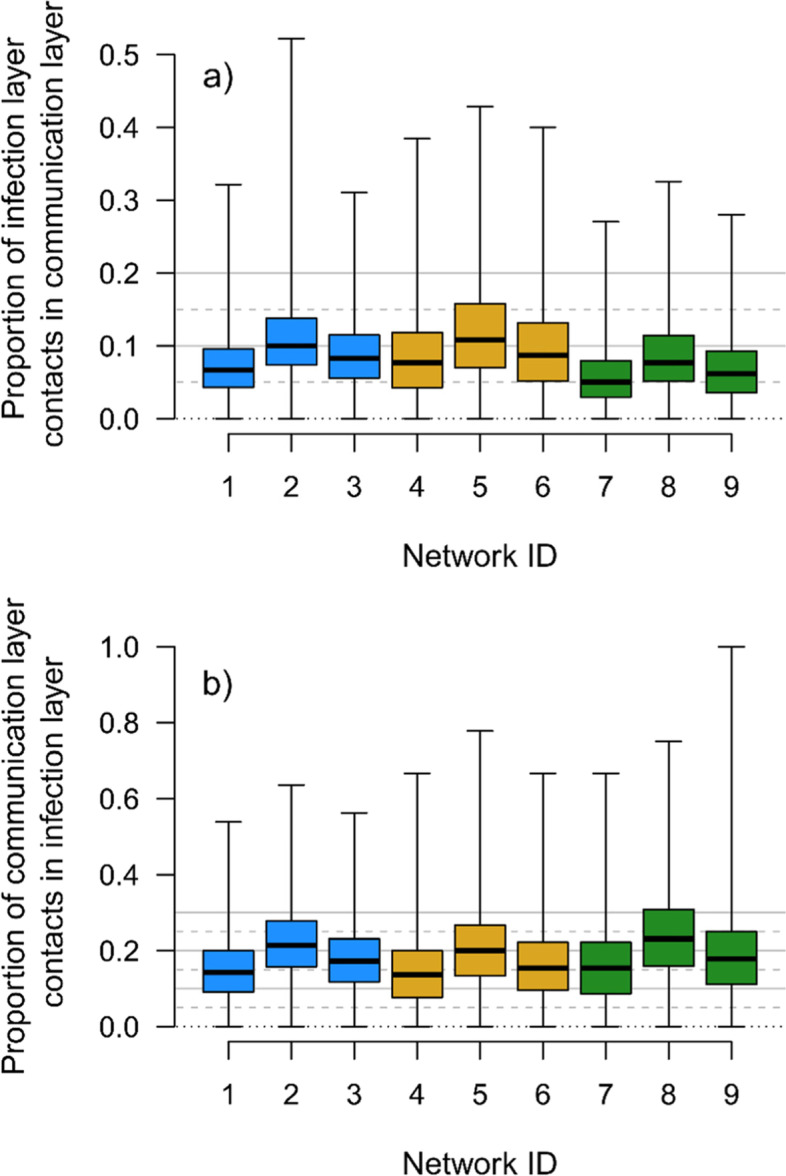
The proportion of contacts in each layer of the multiplex network that are present in the other layer. Panel **a**) shows the proportion of infection layer contacts also present in the communication layer and panel **b**) show the proportion of communication layer contacts also present in the infection layer. Networks 1-3 have no homophily in either layer, networks 4-6 have homophily in both layers and networks 7-9 have homophily in the communication layer only. In networks 1, 4 and 7 both layers have a relative modularity of 0.4, in networks 2, 5 and 8 both layers have a relative modularity of 0.6, and in networks 3, 6 and 9 the relative modularity of the infection layer is 0.6 and the relative modularity of the communication layer is 0.4

The structure of the network was relatively unimportant in determining the success with which populations were able to “flatten the curve” (Fig. 5, Fig. S1). Most strikingly, there was a small negative impact on the value of information from the communication layer when there was homophily only in that layer and not in the infection layer (networks 7-9). When this was the case epidemic peaks remained higher when individuals acquired information on local prevalence from their communication layer. This pattern was driven by their being a greater mismatch between the two layers when there was only homophily in the communication layer; a lower proportion of edges in the infection layer were also present in the communication layer (Fig. 6). It is harder, therefore, to flatten the curve when key aspects of structure of communication and infection layers are mismatched and individuals gain information on illness from their communication layer. Otherwise there were no clear and consistent patterns related to network structure over the range of structures tested here. Results were qualitatively similar regardless of the strength of the Awareness Effect (Fig. S1).

## Discussion

Our study helps illustrate that building a perception of infection risk using trusted social contacts can drive community-level patterns of protective behaviours against disease outbreaks, but only plays a substantial role when there is limited information available about the illness of close physical contacts. Accurate information on prevalence in an individual’s likely physical contacts is very effective in enabling individuals to construct a reliable perception of their risk of infection and so flatten the epidemic curve when this leads to behaviour change. However, the advantage brought about by an individual’s knowledge of the prevalence in their “infection layer” declines very rapidly as the accuracy of this information deteriorates. Homophily in the network can compromise the ability of communities to respond to the actual risk present, especially when it generates mismatches between network layers (most strikingly when the alternative is accurate information on likely physical contacts). These results have strong, direct implications for individuals living in circumstances in which their physical contacts are likely removed from their social spheres of influence. Critically, this pattern reflects large urban centres in which individuals may physically contact many people using public transportation, or riding elevators and moving among offices or apartments in large high-rise buildings, but are likely instead to rely on a mostly separate community of family, faith, or recreational activities for social community and conversation from which they will form their perceptions of risks and norms.

Of course, this main result relies on the low overlap between contacts present in both the communication and disease layers that shape an individual’s perceptions and risks (see Fig. 6). If those layers were instead identical (as in small, remote rural communities or highly segregated, small, well-mixed communities as exist affiliated with some religious groups), then the communication layer and infection layer will be equivalent in the information they provide, meaning that more information is available on local prevalence in an individual’s infection layer and so improving the decision-making of individuals based on observations of their personal networks.

Our results show that the availability of accurate information from an individual’s infection layer is much more effective in mitigating disease spread (i.e. “flattening the curve”) than using only their communication layer, and that when this is the case using information from both only performs marginally better than using the infection layer alone. However, as the availability of accurate information from the infection layer declines, the success of mitigation declines very rapidly. In our networks, a > 20% chance of detecting each symptomatic neighbour in the infection layer is required for mitigation to be more successful than when individuals use their communication layer alone.

The former result suggests that populations comprised of individuals who tend more towards independent risk assessment than towards reliance on community leadership may respond better to public health interventions. However, the latter result indicates the importance of highly accurate information from the infection layer at a community level. Therefore, any social norms that reduce observability of infection in a local community can undercut the efficacy of recommended behavioural interventions. This is especially important in shaping public messaging since both within group density (i.e. modularity) and closeness of beliefs within a community (i.e. homophily) can have less of an impact than the information on which the members of that community rely (Fig. 5). Finding ways to provide people more accurate information on infection prevalence among their likely contacts becomes even more important if people are using this information to gauge their personal risk and adjust their behaviour accordingly.

We therefore strongly support the adoption of public reports of identified cases in local communities that come into regular potential contact with each other. While this can be challenging to achieve in many societies, and requires sensitivity to personal privacy, regular announcements/reminders at a city, company, school, or neighbourhood level of active disease prevalence can potentially provide critical and effective reinforcement for the individual adoption of behaviours that can protect everyone. One well-established route to providing accurate information about contacts in the infection layer is through manual or digital contact-tracing. These approaches are known to be highly effective in mitigating COVID-19 outbreaks [39–42], but can be limited by resources or by uptake [43, 44]. However, a further alternative is to provide accessible, general information about potential exposure locations. For example, this has formed an integral part of Nova Scotia’s effective public health strategy during the current pandemic [45]. It may be that when sufficiently publicised (e.g. through conventional and social media), information on exposure locations is effective through both helping people identify their own potential exposures and also helping people who didn’t visit these locations build a more complete perception of their risk of infection that is not apparent through their own social (communication) ties. The latter could mean these approaches provide a clear additional benefit to using contact tracing alone where information is (necessarily) less publicly available.

Awareness itself is not without complexity. The centralized collection and analysis of data at regional or national scales involves logistical challenges and can cause delay in reporting that information back to the public [46]. It is also frequently the case that communities pay more attention to, and place greater trust in, local sources of information than in more remote sources [47]. Policies that focus on community leadership to ensure a local focus for awareness helps to address both of these difficulties. Our study highlights the need for leaders of social groups to ensure attention is paid to cases of COVID-19 in their community. Their actions can have a positive impact both through providing more accurate information on prevalence within an individual’s infection layer and by helping to prevent misperceptions of risk through mismatched layers in an individual’s social network. They are also likely to act as influential others that play a disproportionate role in shaping a community’s social norms around protective behaviours (e.g. “mavens” [18, 48–50];). Luckily, this is in keeping with the mission of many social groups focused on community support. Communities of worship, social action organizations, and community volunteer groups have all been active participants across the globe in making sure that individuals who are unwell but not so severely impacted as to be hospitalized have access to groceries, medicines, and wellness checks. By actively highlighting the need for these services within their own community, these actions themselves support broader adoption of preventative behaviours and thereby not only help individuals already affected by COVID-19, but actively decrease the likely magnitude of local impacts from the pandemic.

A further complication is provided by other learning processes (e.g. the Social Construction and Reassurance Effects modelled in our study) that may influence each individual’s behaviour. Our model shows the potential importance of perceived social norms in helping to elevate concern and promote adherence with protective behaviours. When social construction of concern was strong there was a more substantial reduction in epidemic peak, even when individuals learned about their infection risk from their communication layer. However, misperceptions of social norms related to health behaviours have been widely documented, and could potentially impact health behaviour [51, 52]. Misperceptions about social norms around protective behaviours in the context of COVID-19 (or other respiratory pathogens) might be expected for various reasons [52]. For example, a common protective behaviour is for people to stay at home (and reduce social contacts). However, once they have adopted these behaviours these adherent citizens are less likely to be encountered by others which could lead to others underestimating concern in their community [52]. Our models also showed that people relaxing their concern from having their social ties in the communication layer healthy impacted the relative value of perceiving infection risk from these same contacts. When the Reassurance Effect was stronger, perceiving direct risk from the communication layer became less effective, especially when individuals played close attention to social norms. When individuals had accurate information from their infection layer instead then the strength of the Reassurance Effect was much less important, indicating that the types of approaches to provide this information discussed above can be important in counteracting erosion in concern over time.

While our model provides valuable insights into the influence of how individuals form their perception of risk on epidemic dynamics, we (necessarily) make a number of simplifying assumptions that are important to take into account when interpreting the results. First, we assume that individual adherence to protective behaviours is binary at any one point in time. In reality, non-pharmaceutical interventions consist of a diversity of protective behaviours and if individuals vary in their adherence to different ones it may complicate these results [33–35]. Second, while we generated plausible multiplex networks applying these types of modelling approaches will be further enhanced by applying them to data-driven network structures. Empirical data on human contact networks has become available during the current pandemic [53, 54] but the ability to combine this with information on other social ties (i.e. the communication layer) remains a major challenge. Third we made a number of assumptions about how people perceive their risk of infection, respond to information about social norms and what causes reductions in concern over time. The availability of more empirical data related to protective behaviours and behaviour change from the current pandemic (e.g. [55–58]) can be used to better develop these components of the model and select appropriate theoretical models of behaviour change [10].

## Conclusions

One of the most fundamental challenges in creating effective public health policies is the design of recommendations that will not only achieve theoretical outcomes but will be adopted by enough of a willing public to accomplish those outcomes in the real world. Integrating an understanding of how individual perceptions shape behaviours, and how social context itself shapes perceptions has become one of the critical stumbling points in our local, national, and global response to the COVID-19 pandemic. Our results clearly show that local, accurate, rapid, and trusted information can enable better emergent behaviours. Thankfully, these paths are within the capability of our public health community and local community leadership to provide.

## Data Availability

Data sharing is not applicable to this article as no datasets were generated or analysed during the current study. However, the relevant code to reproduce the study is publicly available at (https://github.com/matthewsilk/CoupledDynamics2_layeruse).
